# Mapping High‐Rate Clusters of Animal Contact‐Related Human *Salmonella enterica* Single‐State Outbreaks in the United States, 2009–2022: A Spatial Epidemiological Approach to Inform Public Health Surveillance

**DOI:** 10.1111/zph.70071

**Published:** 2026-06-12

**Authors:** Hammad Ur Rehman Bajwa, Suman Bhowmick, Csaba Varga

**Affiliations:** ^1^ Department of Pathobiology College of Veterinary Medicine, University of Illinois Urbana‐Champaign Urbana Illinois USA; ^2^ Carl R. Woese Institute for Genomic Biology University of Illinois Urbana‐Champaign Urbana Illinois USA

**Keywords:** animal contact, incidence, outbreaks, public health, *Salmonella*, single‐state, space–time, spatial, United States

## Abstract

**Introduction:**

Nontyphoidal *Salmonella enterica* (NTS) is a major zoonotic enteric pathogen. Animal contact‐related NTS outbreaks have increased in the United States over the last decade. Geospatial analysis can identify locations with elevated risk of NTS outbreaks where public health authorities can focus their NTS prevention and intervention efforts.

**Methods:**

We analysed NTS outbreak data reported from individual states to the Centers for Disease Control via the National Outbreak Reporting System between 2009 and 2022 across the continental contiguous United States. A geospatial analytical framework that included disease mapping, spatial interpolation, and global and local clustering methods was applied to identify regions with high NTS outbreak rates. Given that the study period (2009–2022) included the COVID‐19 pandemic, an interrupted time series negative binomial model was used to assess changes in NTS incidence before and after 2020.

**Results:**

A total of 104 NTS single‐state outbreaks were reported to the National Outbreak Reporting System (NORS) during the study period. The mean annual incidence rate was 0.02 NTS outbreaks per million person‐years. The primary animal contact categories associated with outbreaks were mammals (cattle, pigs, sheep, and horses), birds (backyard chickens, ducklings, and turkeys), and reptiles (turtles and lizards). Exposure settings included farms, fairgrounds, agricultural feed stores, veterinary clinics, dairy/agricultural settings, and residential settings. The local cluster detection methods consistently identified areas with significantly high NTS animal contact‐related outbreak rates in the Mountain West, Midwest, and Northeast of the US. The interrupted time series analysis indicated a reduction in incidence following the onset of the COVID‐19 pandemic (IRR = 0.03; *p* = 0.06).

**Conclusion:**

NTS animal contact‐related single‐state outbreaks revealed distinct spatial clustering across the United States, with higher risks in the Mountain West, Midwest, and Northeast. Diversity of animal‐contact sources and exposure settings depicted complex transmission dynamics of NTS. A decline in reported NTS outbreaks was observed after the COVID‐19 pandemic. Focused prevention and control programs are needed in high‐risk areas to mitigate the burden of NTS outbreaks.

## Impacts

- Outbreaks clustered in the Mountain West, Midwest, and Northeast regions.
- The pandemic period was associated with a decline in outbreak incidence.
- The most common animals linked to outbreaks were mammals, birds, and reptiles.
- Main exposure settings included farms, veterinary clinics, feed stores, shelters, schools, and homes.
- Region‐specific prevention and interventions are needed to reduce the burden of illnesses.

## 1 Introduction

Nontyphoidal *Salmonella enterica* (NTS) are an important bacterial enteric pathogen that impacts human health (Majowicz et al. 2010). It includes more than 2500 serovars (Lamichhane et al. 2024); however, only a few dominant serovars, including *S. Typhimurium* , *S. enteritidis* , and the monophasic variant *S*. I 4,[5],12:i:, account for most human infections (Hagedoorn et al. 2024). They also show distinct epidemiologic patterns, serovar Enteritidis primarily linked to poultry and Typhimurium associated with multiple animal reservoirs (e.g., livestock, poultry, and reptiles), and both can be transmitted via food and direct animal contact (Bajwa et al. 2026a).

Globally, NTS are a leading cause of gastroenteric infections, with an estimated 153.1 million cases and 56,969 deaths (Kirk et al. 2015), and are primarily transmitted through the consumption of contaminated food products; however, animal contact‐related NTS outbreaks are also significant. Animal contact‐associated salmonellosis occurs through direct contact with infected animals or indirect contact with their contaminated environment (Hoelzer et al. 2011).

Since NTS are zoonotic pathogens with diverse animal reservoirs, they may exhibit regional variation in transmission patterns and disease burden (Yates et al. 2025). Regional differences may exist in agricultural practices, livestock densities, animal‐contact exposure types, urbanization, environmental factors, and demographic and socioeconomic characteristics of human populations, which can influence NTS distribution (Varga et al. 2013a, 2013b; John et al. 2025; Yates et al. 2025). Understanding how these factors impact NTS clustering is important to designing local public health prevention and intervention programs.

Spatial analysis is useful in understanding the NTS distribution across space and time, identifying high‐risk clusters, and assessing risk factors (Seixas et al. 2018). Several studies have been conducted to understand the distribution and clustering of NTS. In Italy, NTS cases clustered in the southern part of the country, where the livestock population was lower, suggesting a non‐livestock environmental NTS reservoir (Graziani et al. 2015). Similarly, in the Netherlands, NTS incidence was not spatially associated with exposure to livestock, suggesting that environmental exposure and foodborne transmission might be the main exposure sources (Mulder et al. 2024). A study in Portugal found persistent urban clusters, highlighting the importance of demographic factors in shaping NTS transmission (Seixas et al. 2018). In Canada, studies have examined the impact of socioeconomic (Varga et al. 2013a; John et al. 2025) and demographic factors (Varga et al. 2013b), and living close to a slaughterhouse (Paphitis et al. 2021) on NTS incidence. Other Canadian studies assessed spatial–temporal clustering of NTS infections, identifying high‐rate clusters during warmer months and highlighting regional variability in infection rates (Varga et al. 2015; Valcour et al. 2016; John et al. 2024).

In the United States, the Centers for Disease Control and Prevention (CDC) coordinates national enteric disease surveillance through the National Outbreak Reporting System (NORS), which monitors outbreaks reported by state, local, and territorial public health agencies (CDC 2023). Previous research using NORS data has compared the characteristics of foodborne and animal‐contact‐related NTS outbreaks, identifying that children are more affected in animal‐contact‐related outbreaks. These NTS outbreaks were often linked to contact with infected backyard poultry, petting zoos, or reptiles (Marus et al. 2019). Other national‐level analyses found that NTS was the second most reported enteric pathogen and had the second highest hospitalization rates among outbreak‐associated infections (Wikswo et al. 2022). In addition, a longitudinal national study of foodborne *Campylobacter* outbreaks in the United States from 1998 to 2016 revealed regional differences in their incidence and exposure sources (Sher et al. 2021). A Florida‐based study identified persistent high NTS infection incidence areas in the northern part of the state (Li et al. 2021).

Despite the extensive literature on NTS infections, recent studies from the United States have focused on specific NTS serovars, short study periods, or narrowly defined geographic regions. A gap remains in national‐level analyses examining longitudinal spatial and spatio‐temporal clustering of animal‐contact‐associated NTS outbreaks.

To address this knowledge gap, the present study uses the data from the NORS to apply a geospatial analytical framework and assess the distribution and clustering of animal‐contact‐related single‐state and multi‐state NTS outbreaks reported at the state level in the United States (US) from 2009 to 2022. In addition, interrupted time series analysis will be used to evaluate temporal trends in outbreak incidence before and after the onset of the COVID‐19 pandemic. Findings from this study can inform regionally focused public health strategies aimed at reducing zoonotic transmission of NTS and provide a model for analysing other enteric pathogens.

## 2 Materials and Methods

### 2.1 Study Setting

All animal contact‐related NTS outbreak information that was reported to the CDC via NORS between 2009 and 2022 was obtained through a data request. Single‐state outbreaks, where exposures occurred in a single state, were included, and because of the geospatial analyses, we included data from the contiguous United States, which consists of 48 states and the District of Columbia that share a land border (Figure S1). An outbreak was defined as the occurrence of a greater‐than‐expected number of cases within a specific period, specific location, and a target population (Reintjes and Zanuzdana 2009). State‐level population data for the study period were obtained from the US Census Bureau (US Census Bureau 2024a). The administrative boundary shapefile was obtained from the TIGER/Line database (US Census Bureau 2024b). For all spatial analysis, the shapefile was projected to the USA Contiguous Albers Equal Area Conic USGS, as it is well‐suited for areas extending in an east‐to‐west orientation (Esri 2025).

### 2.2 Data Analysis

The data cleaning, management, and descriptive statistics were performed using the R statistical software (version 4.1.2 (2021‐11‐01)) (R Core Team 2024). The spatial analyses were conducted in ArcGIS Pro 10.7.1 (Environmental Systems Research Institute Inc., Redlands, CA, USA).

#### 2.2.1 Mapping Nontyphoidal *Salmonella* Single‐State Outbreak Incidence Rates

In the first step, state‐level NTS single‐state outbreak mean incidence rates (IRs) per 1 million population‐years (1 MPY) were calculated for two periods (2009–2015 and 2016–2022) by dividing the number of NTS outbreaks in each state in each period by the corresponding state and period‐specific population and multiplying it by 1 million. The state‐level mean IRs per 1 MPY for the two periods were illustrated in choropleth maps, using the natural breaks classification with 5 breaks (Jenks 1967). The categorization was slightly changed to include a class representing an IR of 0.

In the next step, to account for unstable IRs of states with small populations, the spatial empirical Bayes (SEB) smoothing method was used (Marshall 1991) in GeoDa software (Anselin et al. 2006). For spatial weighting, the 1st‐order Queen contiguity criterion was used, which defines the neighbours as spatial units with a shared vertex or edge, and considers the immediate neighbours (Anselin 1995). The distribution of raw (non‐smoothed) and SEB‐smoothed NTS outbreak IRs was illustrated in choropleth maps.

In the last step, empirical Bayes kriging (EBK) (Gribov and Krivoruchko 2020) was used to spatially interpolate the raw NTS IRs for the two study periods, which were illustrated in isopleth maps. The EBK method estimates IRs empirically from the existing data and averages IR predictions over multiple simulations to capture uncertainty in the data (Krivoruchko and Gribov 2019).

#### 2.2.2 Global Cluster Analysis

For the two time periods, incremental spatial autocorrelation analysis (ISA) using Moran's I method estimated global clustering of NTS IRs over a series of increasing incremental Euclidean distances (Mitchell 1999). For each distance increment, *z*‐scores and *p*‐values were calculated and illustrated in a graph, and the highest *z*‐score represented the maximum peak, where spatial processes promoting spatial clustering were more active (Mitchell 2021). The distance band associated with the highest z‐score was selected for the subsequent local cluster analyses.

#### 2.2.3 Local Cluster Analysis

Each state was represented by a polygon, its centroid, and the corresponding IR for the respective study periods (2009–2015 and 2016–2022). For the conceptualization of spatial relationships, the “zone of indifference” parameter, which assigned equal spatial weights to states within the specified threshold distance around the index area, while this weight decreased as that distance was passed, allowed for a gradual decay of spatial influence (Mitchell 1999).

##### 2.2.3.1 Hot Spot Analysis (Getis‐Ord Gi* Statistics)

The hotspot analysis identified statistically significant (*p* ≤ 0.05) clusters of high or low NTS outbreak IRs. The statistically significant high *z*‐score depicted a hot spot, where a high IR state was surrounded by high IR states, a low *z*‐score depicted a cold spot, where a state with low IR was surrounded by low IR states, while an almost zero z‐score revealed no significant clustering and implied spatially uniform distribution of risk (Getis and Ord 1992). Each state‐level cluster was classified into hot spot and cold spot categories based on their statistical significance level, 99% confidence (*p* ≤ 0.01), 95% confidence (*p* ≤ 0.05), 90% confidence (*p* ≤ 0.10), and non‐significant (*p* > 0.10).

##### 2.2.3.2 Cluster and Outlier Analysis (Anselin's Local Moran's I)

This method detects clusters with similar IRs and spatial outliers where IRs differ significantly from the neighbouring states. Four cluster types are identified: High‐High (high IR state surrounded by High IR states), Low‐Low (low IR state surrounded by low IR states), High‐Low (a high IR state surrounded by low IR states), and Low‐High outlier (low IR state surrounded by high IR states) (Anselin 1995).

#### 2.2.4 Scan Statistics

Scan statistics were conducted using the SaTScan v10.3.2 software (Kulldorff 2022) to identify purely spatial and space–time clusters of NTS single‐state outbreaks across the US during the whole study period from 2009 to 2022. The retrospective discrete Poisson model was applied (Kulldorff 1997). For the spatial scan analysis, the circular scanning window was set to include up to 10% of the population at risk (Kulldorff 2022), which searched for high or low NTS IR clusters. The retrospective space–time analysis included a cylindrical scanning window (a geographic base with a temporal interval height) that searched for high or low rate NTS IR clusters. The maximum spatial cluster size was set to include 10% of the population at risk and 50% of the study period. The relative risk (RR) for each identified cluster was estimated as the ratio of observed to expected outbreaks within the cluster compared with outside the cluster (Kulldorff 2022). The RR was calculated as,

$$\mathrm{RR}=\frac{o/e}{\left(T-o\right)/\left(T-e\right)}$$

where o was the total number of NTS single‐state outbreaks in a state, e was the total number of expected NTS single‐state outbreaks in a state, T was the total number of observed NTS single‐state outbreaks in the United States.

An RR greater than 1 indicated a higher‐than‐expected, while RR < 1 reflected a lower‐than‐expected NTS outbreak (Kulldorff 2022). To avoid assuming identical risk within a significant cluster, the RR for each area included in the cluster was calculated and mapped. The statistical significance was determined using Monte Carlo hypothesis testing with 999 replications (Kulldorff 2022).

#### 2.2.5 Interrupted Time Series Analysis of NTS Outbreak Trends Pre‐ and Post—COVID‐19

A generalized linear model with a log link and Poisson distribution was constructed. The number of outbreaks in each year was the outcome, and the logarithm of the population was the offset. The model incorporated terms for time (to estimate the underlying pre‐pandemic trend), an indicator variable for the COVID‐19 period (post), and a post‐period time variable (time_post) to capture changes in slope following the onset of the pandemic. The model structure was:

$$Y_{t}∼\text{Poisson}\left(\mu_{t}\;\right)$$

$$\log\left(\mu_{t}\right)=\beta_{0}+\beta_{1}\cdot \mathrm{tim}e_{t}+\beta_{2}\cdot \mathrm{pos}t_{t}+\beta_{3}\cdot \text{time}\_\mathrm{pos}t_{t}+\log\left(\text{Populatio}n_{t}\right)$$

In the presence of overdispersion, a negative binomial model was applied. Counterfactual predictions were obtained by extrapolating the pre‐pandemic trend into the post‐2020 period, assuming no change associated with the COVID‐19 period. In addition, the overall percentage change in incidence rates between the pre‐pandemic and pandemic periods was calculated based on mean incidence rates.

## 3 Results

### 3.1 Mapping Raw NTS Outbreak Incidence Rates

**FIGURE 1:**
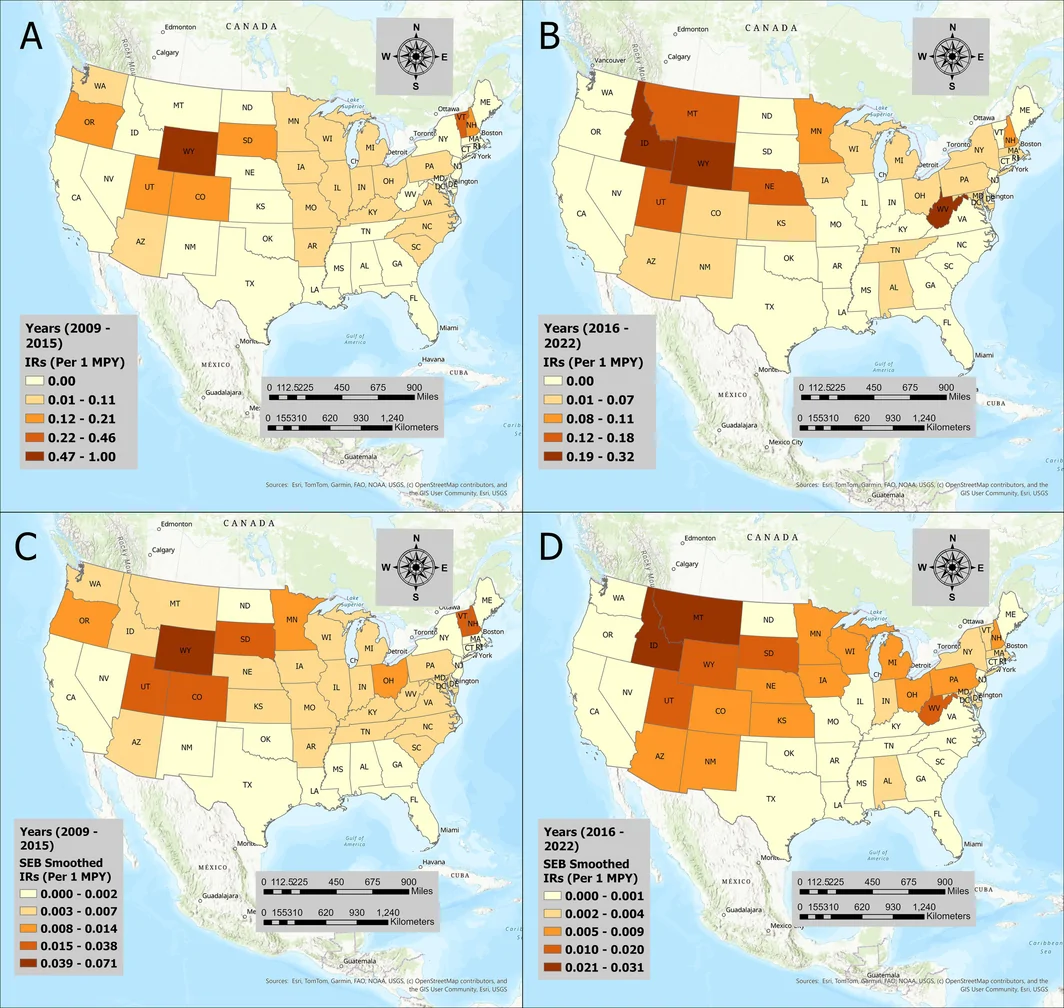
Incidence rates (per 1 million population‐years) of single‐state outbreaks across the United States for two periods (2009–2015, and 2016–2022) (Raw incidence rates: A and B; Smoothed incidence rates: C and D). The spatial empirical Bayes smoothing with first‐order queen contiguity weights was used to smooth the incidence rates.

Figure 1A,B illustrates the state‐level raw IRs of NTS outbreaks for the two study periods (2009–2015 and 2016–2022) in choropleth maps.

In the first period, Wyoming reported the highest IR (1.00 per 1MPY), followed by Vermont (0.46), New Hampshire (0.21), Alaska (0.20), Utah (0.20), South Dakota (0.18), and Colorado (0.16). The states with moderate to low IRs were Arkansas (0.05), Minnesota (0.05), Michigan (0.03), Arizona (0.02), and Illinois and North Carolina (0.01). The states that had no reported outbreaks during this period were Alabama, Hawaii, Idaho, Kansas, Montana, New Mexico, Tennessee, Texas, and West Virginia (Figure 1A,B; Table S1).

During the second period, Idaho reported the highest IR (0.32 per 1MPY), followed by Wyoming (0.25), West Virginia (0.24), Utah (0.18), Nebraska (0.15), Montana (0.14), New Hampshire (0.11), Hawaii (0.10), and Wisconsin (0.05). The states with moderate to low IRs were Michigan (0.04), Colorado (0.03), Maryland (0.02), and New York (0.01). The states reported no outbreaks during this period were Alaska, Kentucky, Oregon, Arkansas, Illinois, Missouri, and Virginia (Figure 1A,B; Table S1).

Overall, higher outbreak incidence rates were observed during the first period (2009–2015), with the highest rates concentrated in the Mountain West, while parts of the Northeast and Southern regions reported low to no outbreaks. This spatial distribution remained consistent in the second period, although overall rates were lower.

### 3.2 Mapping Smoothed NTS Outbreak Incidence Rates

The NTS outbreaks' SEB smoothed IRs in each state were presented in choropleth maps for the two study periods (2009–2015 and 2016–2022) (Figure 1C,D; Table S2). In the first period (2009–2015), Wyoming reported the highest IR of 0.070 per million population‐years, followed by Vermont (0.03) and Utah (0.02). The states with the lowest IRs were Nebraska (0.01), New Mexico (0.006), New York (0.002), and Texas (0.0008). In the period 2016–2022, Utah reported the highest IR of 0.02, followed by Wyoming (0.01), Minnesota (0.009), and Ohio (0.006). The states with the lowest IRs were Texas (0.0008), Oregon (0.0007), and Virginia and Washington (0.0004) (Figure 1C,D; Table S2). Overall, the Mountain West and Northeast regions continued to show elevated SEB smoothed outbreak IRs, indicating persistently high‐rate regions.

### 3.3 Empirical Bayes Kriging of NTS Outbreak Incidence Rates

**FIGURE 2:**
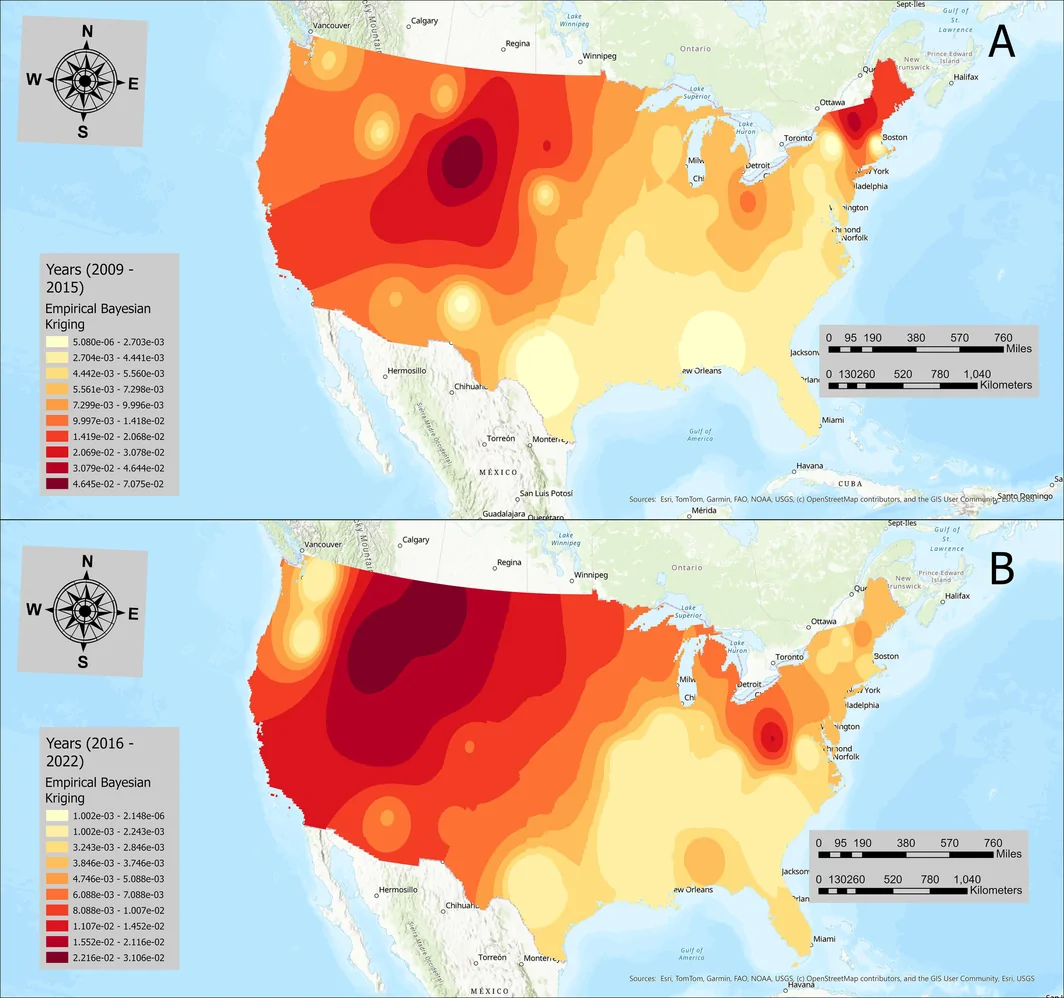
Spatial interpolation of outbreak incidence rates for two periods (2009–2015, and 2016–2022) across the United States. The empirical Bayesian kriging method was used to interpolate the outbreak incidence rates (outbreaks per 1 million person‐years).

Figure 2 illustrates the spatially interpolated IR values in isopleth maps for the two study periods.

During 2009–2015, the elevated interpolated values (0.020–0.046) were observed in the Mountain West region. Moderate to low values were observed in the upper Northeast and upper Midwest regions (0.0045–0.020). The lowest interpolated values were in the Southern and Southwestern regions.

During the second period (2016–2022), the Mountain West remained a high IR region, albeit with lower values than in the first period. In addition, parts of the upper Northeast emerged as high‐interpolated value regions (0.011–0.0031), and this trend further radiated towards the Pacific midwestern region. Moderate to low values were observed in the Central Plains and the lower Northeastern region (0.005–0.011), while various parts of the Southern region were consistently lowest (Figure 2).

### 3.4 Global Clustering

**FIGURE 3:**
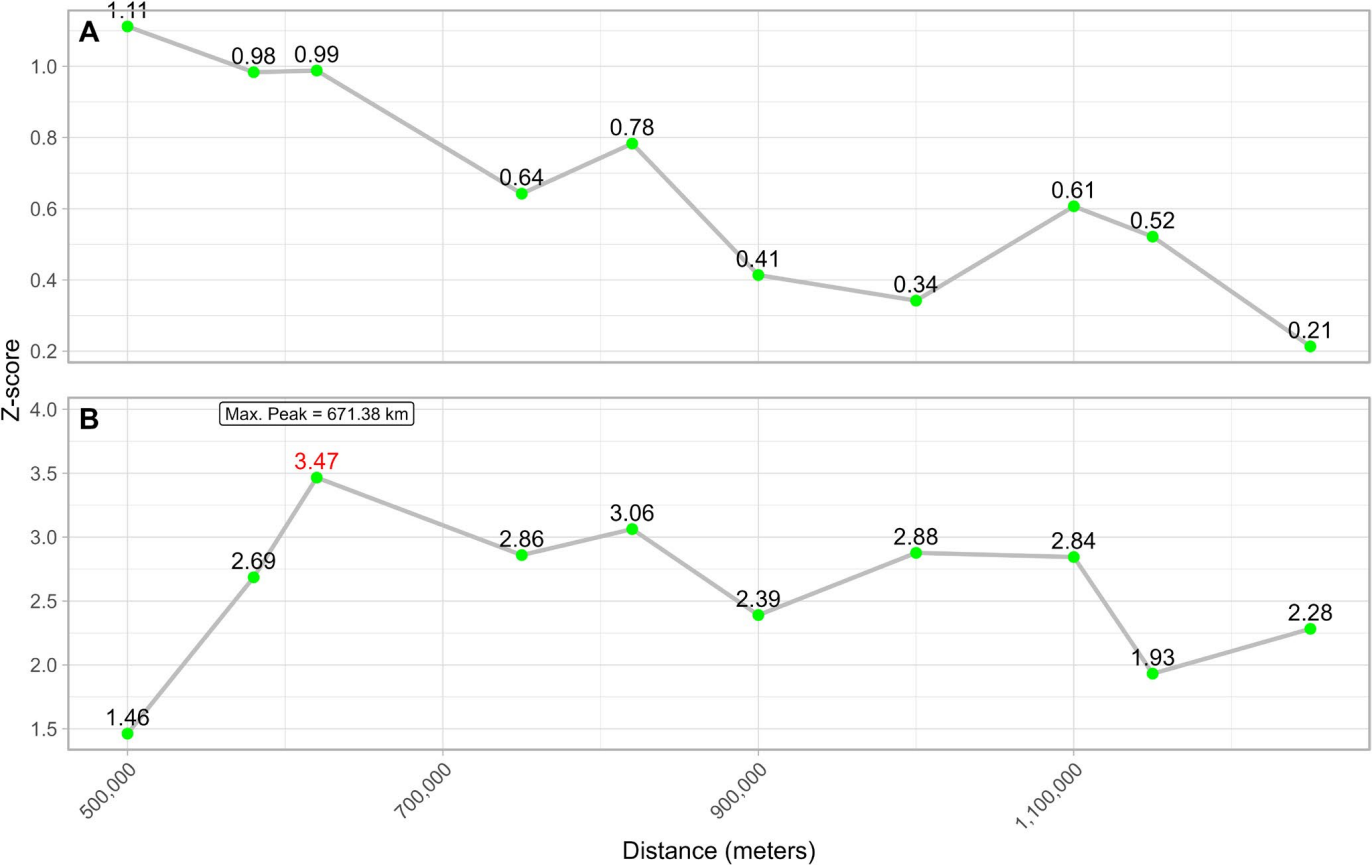
Incremental spatial autocorrelation analysis of outbreak incidence rates for two periods (2009–2015, and 2016–2022) across the United States. Ten distance increments were used with the fixed‐distance band to conceptualize spatial relationships. Significant at *p* ≤ 0.05, *Z*‐score ≥ 1.96.

The incremental autocorrelation analysis assessed the global clustering of NTS IRs across the US for two study periods: 2009–2015 and 2016–2022 (Figure 3A,B).

The analysis estimated significant spatial autocorrelation across 10 incremental distances using global Moran's *I* statistic. In 2009–2015, no significant peak was observed (Figure 3A; Table S3). While in the period 2016–2022, the first and maximum peak was observed at 671.38 km (Moran's *I* = 0.252, *z* = 3.46, *p* = 0.001) (Figure 3B; Table S4).

### 3.5 Local Clustering

#### 3.5.1 Hot Spot (Getis‐Ord Gi*) Analysis

**FIGURE 4:**
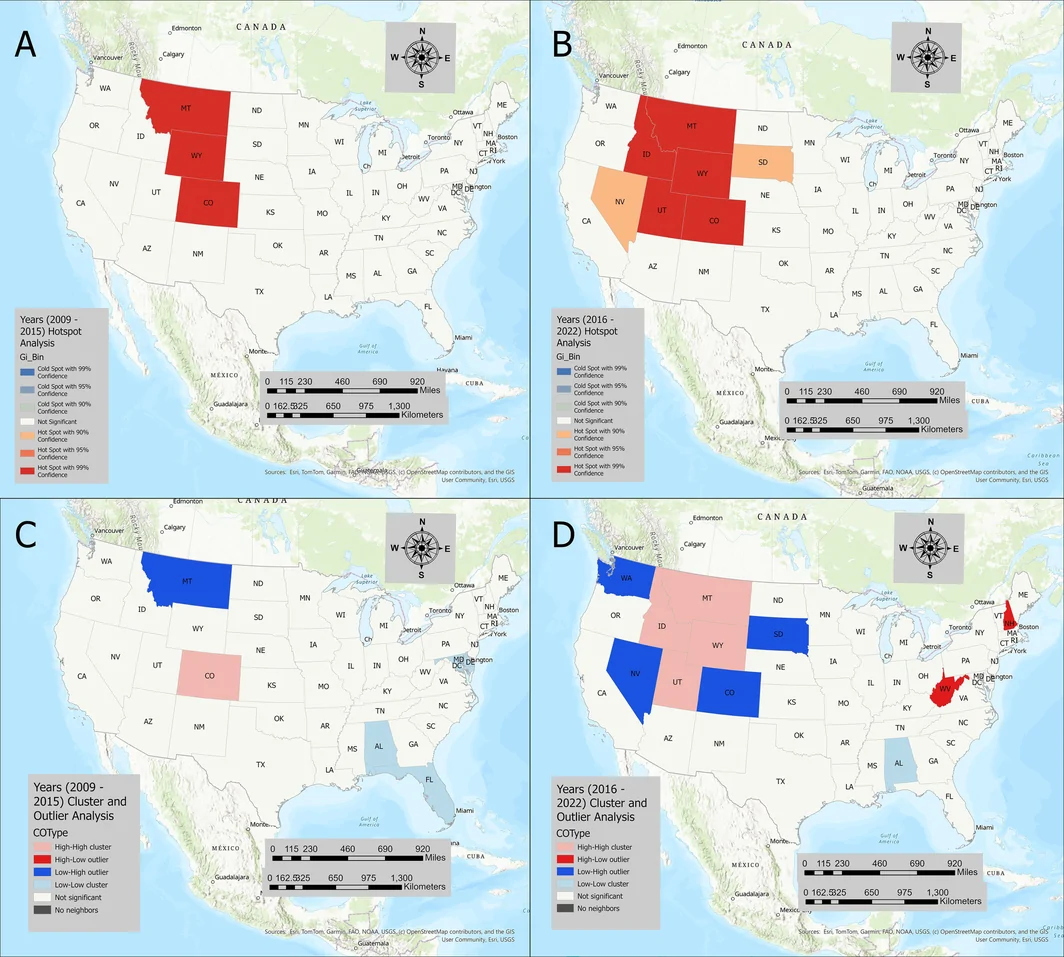
Maps illustrating the results of the local cluster analysis of outbreak incidence rates for two periods (2009–2015, and 2016–2022) across the United States. Hotspot (Getis‐Ord Gi*) analysis (A and B) and Cluster, and Outlier analysis (Anselin Local Moran's *I*) (C and D).

In 2009–2015, significant hotspots were detected in Wyoming, Montana, and Colorado (99% confidence) (Figure 4A).

The animal contact sources that were associated with NTS single‐state outbreaks in these states during this period were baby chicks, ducklings, small turtles, pigs, and small mammalian pets. The most common exposure settings were “Others” (Table S5).

During 2016–2022, Wyoming, Montana, Idaho, Colorado, and Utah were detected as hotspots (99% confidence), followed by Nevada and South Dakota as hotspots with (90% confidence). While no cold spots were detected during this period (Figure 4B). The animal contact sources that were associated with these states during this period were poultry (chicks, ducklings, turkeys, and chickens) and mammals (cattle, pigs, horses, and mice), while the exposure settings were “Others”, fairground, farm/dairy/agricultural settings, and veterinary clinic (Table S5). Collectively, the Getis‐Ord Gi* analysis identified recurrent clustering of elevated outbreak incidence rates in the Mountain West region across both study periods.

#### 3.5.2 Cluster and Outlier Analysis (Anselin Local Moran's *I*)

Figure 4C,D illustrates the significant clusters identified by the local Moran's I statistic. In the first period (2009–2015), significantly high‐high (HH) clusters were identified only in Colorado, and low‐low (LL) clusters were detected in Alabama and Florida. The LH outliers were detected only in Montana; there were no HL outliers during this period (Figure 4C).

The associated animal contact sources linked to NTS outbreaks in these states during this period were baby chicks, ducklings, small turtles, and small mammalian pets. The exposure settings were “Others” (Table S5).

During the second period (2016–2022), HH clusters were found in Montana, Idaho, Utah, and Wyoming, and an LL cluster in Alabama. The HL outliers were detected in West Virginia and New Hampshire. LH outliers were found in Washington, Nevada, Colorado, and South Dakota during this period (Figure 4D; Table S5). The animal contact NTS outbreak exposure sources that were associated with these states during this period were poultry (chicks, ducklings, turkeys, and chickens), mammals (cattle, pigs, sheep, goats, horses, and ferrets), and reptiles (turtles and lizards), while the exposure settings were “Others”, farm/dairy/agricultural settings, agricultural feed store, and Child daycare/preschool (Table S5).

### 3.6 Scan Statistics

**FIGURE 5:**
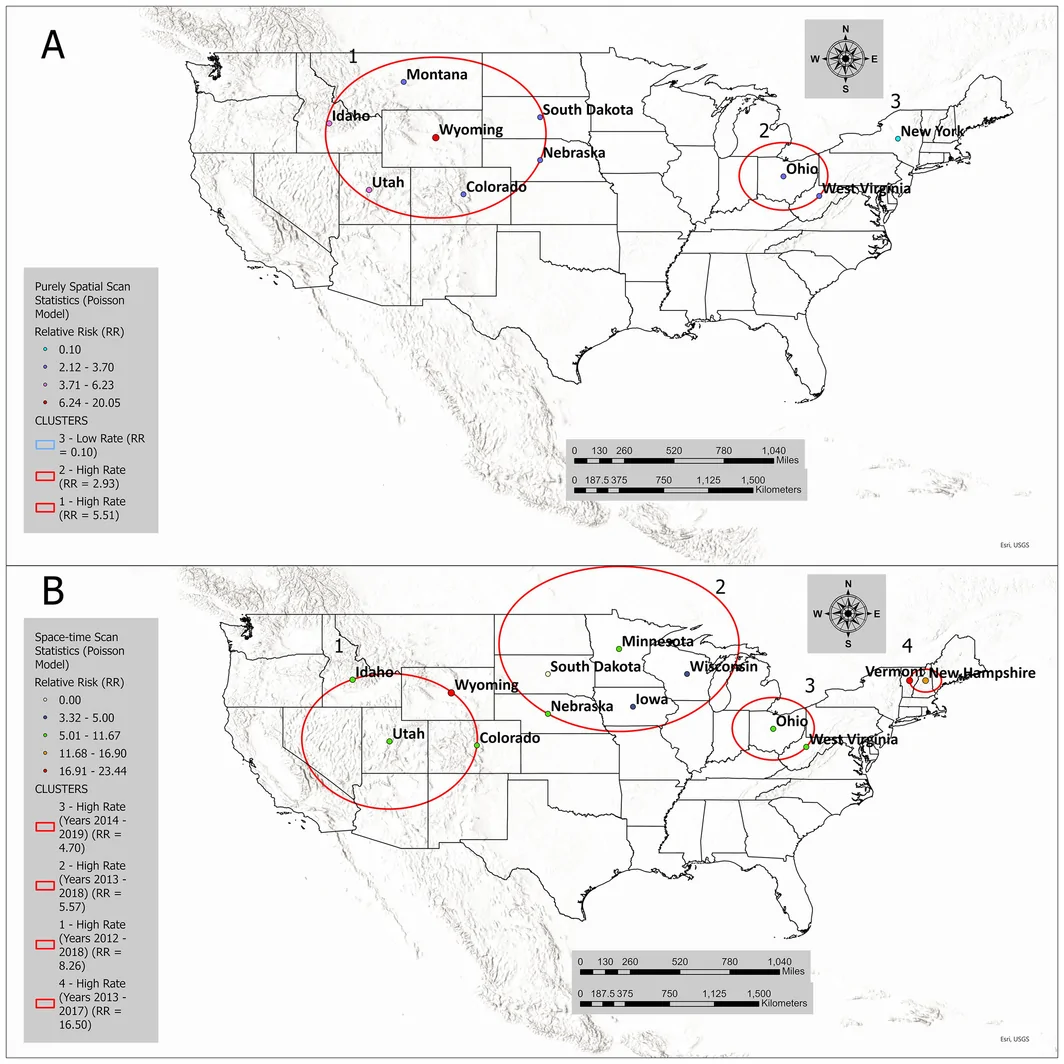
Spatial and space–time clustering of outbreak incidence rates across the United States from 2009 to 2022. (A) Purely spatial scan statistics results. (B) Space–time scan statistics results.

In purely spatial analysis, three significant (Table 1 and Figure 5A) and four nonsignificant (Table S6) spatial clusters were detected using a discrete Poisson model.

**TABLE 1:** Significant spatial and space–time clusters of animal contact‐related NTS outbreaks across the United States, 2009–2022.

Cluster	Model type	# States	Time frame	Population	N/C	E/C	O/E	LR	RR	*p*
1	Purely spatial	7	—	14,580,728	28	6.62	4.23	21.70	5.51	0.001
2	Purely spatial	2	—	13,464,767	16	6.11	2.62	6.05	2.93	0.047
3	Purely spatial	1	—	19,616,550	1	8.90	0.11	6.05	0.10	0.047
1	Space–time	4	2012–2018	10,782,518	17	2.42	7.01	19.69	8.26	0.001
2	Space–time	2	2013–2018	13,464,767	13	2.62	4.97	11.03	5.57	0.005
3	Space–time	3	2014–2019	17,170,774	14	3.35	4.18	9.97	4.70	0.011
4	Space–time	2	2013–2017	1,975,331	5	0.32	15.72	9.20	16.50	0.021

Abbreviations: E/C, expected cases; LR, likelihood ratio; N/C, number of cases; O/E, observed/expected; RR, relative risk.

The primary spatial cluster, with an RR of 5.51, was a high‐rate cluster that included the states of Wyoming, Montana, Idaho, Utah, Colorado, South Dakota, and Nebraska. Animal sources that were associated with these outbreak clusters were birds (baby chicks and ducklings), reptiles (small turtles, lizards, and snakes), and mammals (cattle, sheep, pigs, goats, dogs, and small mammalian household pets [mice, ferret, hedgehog, and guinea pig]). The exposure settings were farm settings, residential settings, and others (Figure 5A; Table S7).

The second spatial cluster was a high‐rate cluster that included two states: Ohio and West Virginia (RR = 2.93). The animal sources that were associated with these outbreak clusters were birds (baby chicks and ducklings), reptiles (small turtle, snake, and other reptiles), pet fish, and mammals (cattle, mice, and hedgehogs). The exposure settings were “Other”, school/college/university, and farm/dairy/agricultural settings (Table S7).

The third spatial cluster was a low‐rate cluster that included the state of New York with an RR of 0.10 (Figure 5A). The animal sources that were associated with this outbreak cluster were reptiles (lizards—bearded dragon). The exposure settings were “Other” (Table S7).

In space–time scan analysis, four significant clusters with high rates (Table 1, Figure 5B) and five non‐significant clusters, one high rate and four low rates (Table S6), were detected using a discrete Poisson model.

The primary high‐rate space–time cluster occurred between 2014 and 2019 and included five states: South Dakota, Nebraska, Minnesota, Iowa, and Wisconsin, with an RR of 4.70. The animal sources that were associated with these outbreak clusters were birds (baby chicks, ducklings, chickens, turkeys, and ducks), reptiles (turtle, roughneck monitor lizard, and bearded dragon), and mammals (cattle, pig, horse, sheep/goat, ferret, hedgehog, mouse, and guinea pig). The exposure settings were educational institutions, veterinary clinics, and “Other” (Table S7).

The second high‐rate space–time cluster occurred during 2013–2018, and included Ohio and West Virginia with an RR of 5.57. The animal sources that were associated with these outbreak clusters were birds (baby chicks and ducklings), mammals (cattle, pigs, dogs, guinea pigs, and hedgehogs), and reptiles (turtles and lizards). The exposure settings were farm settings, child daycare, educational institutions, and “Other” (Table S7).

The third high‐rate spatial cluster was located in Idaho, Colorado, Utah, and Wyoming between 2012 and 2018, with an RR of 8.26. The animal sources that were associated with these outbreak clusters were birds (baby chicks and ducklings), reptiles (lizards (bearded dragon), corn snake, and small turtles), and mammals (dogs, pigs, cattle, sheep/goat, and horse). The exposure settings were farm settings, child daycare/preschool, and a veterinary clinic.

The fourth space–time high‐rate cluster was detected in Vermont and New Hampshire from 2013 to 2017 with an RR of 16.50. The animal sources that were associated with these outbreak clusters were birds (baby chicks and ducklings), mammals (dogs), and reptiles (pet turtles). The exposure settings were farm settings and “Other” (Table S7).

Table S8 summarizes the annual state‐level counts of NTS animal contact–related single‐state outbreaks from 2009 to 2022, including associated animal sources and exposure settings, highlighting temporal variation and geographic distribution of outbreaks across the United States.

### 3.7 Interrupted Time Series Analysis of NTS Outbreak Trends Pre‐ and Post‐COVID‐19

**FIGURE 6:**
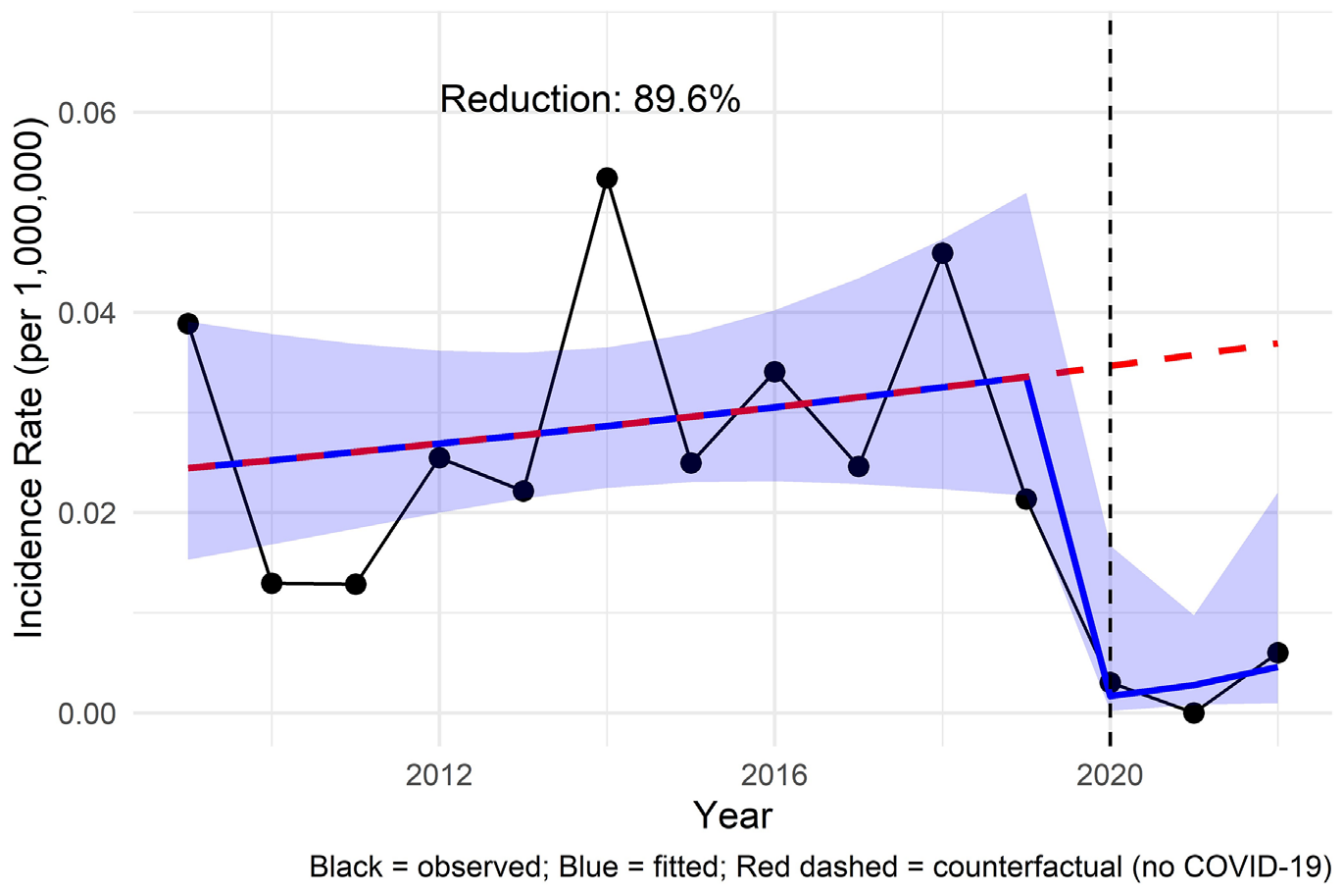
Interrupted time series of outbreak incidence rates (per 1,000,000 population), 2009–2022. Observed data (black), model fit (blue), and counterfactual projection (red dashed) are shown. The vertical line indicates the start of the COVID‐19 period (2020). Incidence declined by 89% during the pandemic period.

The observed outbreak IRs (per 1 MPY) across years ranged from 0 in 2021 to 0.05 in 2014 (Figure 6).

The Poisson model was over‐dispersed (Pearson *χ* ^2^/df = 1.93); therefore, a negative binomial model was used. No significant pre‐pandemic trend in incidence rates (IRR = 1.03, 95% CI: 0.96–1.12, *p* = 0.42) was observed (Figure 6). Following the onset of the COVID‐19 pandemic, incidence declined, with an estimated 97% (IRR = 0.03, 95% CI: 0.0002–0.61, *p* = 0.064). This was consistent with the descriptive analysis, which demonstrated an overall 89% reduction in mean incidence rates between the pre‐pandemic and pandemic periods. No significant change in post‐pandemic trend was observed (IRR = 1.61, 95% CI: 0.38–9.97, *p* = 0.54). Counterfactual analysis indicated that observed post‐2020 incidence remained below the expected trajectory based on pre‐pandemic trends.

## 4 Discussion

This study used a stepwise spatial framework that integrated disease mapping, global and local clustering analyses, and space–time scan statistics to characterize the spatial and temporal patterns of NTS outbreaks linked to animal contact in single states reported to the CDC through the National Outbreak Reporting System (NORS) from 2009 to 2022. We examined the distribution of NTS outbreak incidence rates (IRs), their exposure sources, and settings at the state level across two distinct periods (2009–2015 and 2016–2022). We conducted space–time scan statistics over the entire study period (2009–2022) to identify higher‐than‐expected NTS outbreak rates and assess the persistence and recurrence of outbreak clusters. This integrated approach detected states with consistently elevated risk and linked these spatial patterns to specific animal contact‐related exposure settings and sources. In our analysis, the Mountain region (e.g., Wyoming, Idaho, and Colorado) emerged as a consistent high‐risk area across all spatial analyses. The agreement across the spatial methods suggests the importance of this regional pattern. In addition, we used an interrupted time series analysis to demonstrate a decline in outbreak incidence following the onset of the COVID‐19 pandemic.

Our analysis identified differences in animal type and exposure settings of NTS outbreaks across regions. In the Mountain West region, recurrent NTS outbreaks were linked to contact with cattle, poultry, and reptiles in farms, educational, and residential settings, while the Midwest and Appalachia showed diverse host risk factors, including contact with cattle, poultry, reptiles, and small pets. In the Northeast, outbreaks were associated with reptiles and backyard poultry. By contrast, the South region showed persistently low outbreak clustering. Together, these patterns suggest that animal‐contact‐related NTS outbreaks are influenced by regional animal exposure sources and settings.

In our spatial analysis, global clustering was analysed across two study periods (2009–2015 and 2016–2022). The first period showed no significant global clustering, while the second revealed a peak at 671.38 km, the distance at which the extent of global clustering was the strongest. Considering the average area of single states and their proximity to neighbouring states, this distance likely reflects smaller regional‐scale spatial dependence of outbreaks among adjacent or nearby states rather than broad national‐level global clustering.

The spatial cluster analysis consistently identified the Mountain West region, specifically the states of Wyoming, Montana, and Colorado, as a high‐risk NTS outbreak area. These regions are known for high livestock density (USDA 2024). Cases included in these spatial clusters commonly reported contact with cattle as their exposure source. Infected cattle, whether symptomatic or asymptomatic, can harbour NTS in their gastrointestinal tract, contaminating their faeces and the surrounding environment, which can lead to human exposure through handling livestock or contact with contaminated surfaces, equipment, or water sources (Bentum et al. 2025). Cattle as reservoirs for NTS present challenges for public health, especially in areas with concentrated livestock farming (Arnold et al. 2023). Rural areas with high‐density livestock farms have been described previously as risk factors for the transmission of NTS to humans (Shaw et al. 2016). Also, NTS outbreaks and human exposures often originate on farms with improper biosecurity measures (Kudirkiene et al. 2020), where improper handling of animal waste, inadequate sanitation, and poor management of feed and water sources exist (Koyun et al. 2023). Mitigating the risk of salmonellosis on cattle farms in high‐risk regions identified in our analysis requires developing prevention and control programs, including improved farm management practices, regular health screening of livestock for infections, and educational efforts for both farm workers and the general population to reduce exposure risks (Barnhardt and Raabis 2025; Shrestha et al. 2025).

The Mountain West region is primarily rural, and contact with livestock is not limited to farm workers. Residents could be exposed to animals at fairgrounds, petting zoos, or while visiting a farm. Animals in these settings, including cattle, goats, pigs, sheep, and poultry, can harbour enteric pathogens such as NTS, and they can shed them in their faeces and can also contaminate their environment, leading to human infections through direct contact with infected animals or indirect contact by ingestion of contaminated materials (Conrad et al. 2017; Dunn et al. 2015).

The space–time scan statistics, besides the Mountain West region, identified regions in the Midwest (Ohio, Wisconsin, Iowa, Minnesota, Nebraska) and the Northeast (Vermont and New Hampshire) as high‐risk regions over a relatively long period (2013–2019), suggesting the outbreaks are not isolated incidents but part of sustained risk factors in these areas. In our analysis, these NTS outbreaks were associated with contact with livestock (cattle, horses, and pigs) and poultry (baby chicks, ducklings, chickens, and ducks), and the cases' exposure settings included veterinary clinics, fairgrounds, and farms. This finding could be explained by the high livestock farm density in these regions (USDA 2024), which increases residents' exposure risk to infected animals or to their contaminated environment, resulting in recurring outbreaks or disease occurrences over a long period. These environments could facilitate close human‐animal contact, and residents' lack of awareness of zoonotic transmission potential increases their NTS infection risks (Silva et al. 2014). Also, the Midwest is a central hub for livestock production and processing in the United States, and the movement of animals across state lines can facilitate the spread of pathogens (Cabezas et al. 2021). The recurrent high‐rate space–time clusters in the Mountain West, Midwest, and Northeast indicate that outbreaks occurred repeatedly over time, suggesting the influence of sustained regional exposure factors, such as agricultural practices and animal contact exposures (Kudirkiene et al. 2020).

Besides, livestock and poultry, contact with reptiles was the third most common NTS outbreak source. Reptiles, including turtles, lizards, and snakes, are recognized reservoirs for NTS, and they can be transmitted to humans through direct or indirect contact (Bajwa et al. 2025; Sayers 2022). A recent meta‐analysis estimated the global prevalence of *Salmonella* spp. in reptiles at 30.4%, with snakes at 63.1% having the highest prevalence, followed by lizards (33.6%) and turtles (11.2%) (Muslin et al. 2025).

The study period (2009–2022) included the COVID‐19 pandemic (2020–2022), which likely affected human mobility, social interactions, human‐animal contact, and outbreak reporting. We used an interrupted time series model with a 2020 breakpoint to compare pre‐pandemic and pandemic trends in NTS outbreak incidence, observing a decline during the pandemic, consistent with prior findings of reduced foodborne salmonellosis in the United States (Ray et al. 2021). In addition, no space–time clusters were detected after 2020, which is likely attributable to reduced outbreak detection and reporting during the pandemic.

Before interpreting our study results, a few limitations should be noted. First, the outbreak‐level data included reported animal‐contact exposures; however, because of the ecological design of this study, associations between outbreak clustering, exposure sources, and settings should be interpreted as population‐level patterns rather than individual‐level causal relationships.

Next, our data included NTS outbreaks that were reported voluntarily by state, local, and territorial health departments, and local public health resources could influence reporting (Tilashalski et al. 2022). Additionally, differences in outbreak investigations, surveillance capacity, diagnostic practices, and reporting completeness across states may influence the observed spatial patterns and contribute to regional variation in reported outbreaks (Crane et al. 2022). Furthermore, we only included single‐state NTS outbreaks to assess local exposure sources and settings; however, multistate NTS outbreaks also pose a high public health burden (Eisenstein et al. 2025; Bajwa et al. 2026b), and future studies should assess their impact. Lastly, the COVID‐19 pandemic was likely a confounder for the decreased NTS reports between 2020 and 2023, as local public health resources were reallocated towards addressing the pandemic (Zarella et al. 2024).

## 5 Conclusion

Our study identified persistent spatiotemporal clustering of NTS outbreaks across the US, consistently identifying high‐risk areas in the Mountain West, Midwest, and Northeast of the US. Exposure to cattle, poultry, and reptiles was the most common source of these outbreaks. A multitude of exposure settings, including farms, veterinary clinics, fairgrounds, residential homes, and education facilities, were reported, suggesting that the risk of salmonellosis is elevated in a variety of settings where human‐animal interactions are frequent. Locally focused prevention and control strategies are needed in high‐risk areas, including the development of public education programs on the zoonotic risks of NTS, restrictions on animal contact, effective sanitation in fairgrounds and petting zoos, and regular surveillance to prevent zoonotic infections.

## Funding

The authors have nothing to report.

## Ethics Statement

The authors have nothing to report.

## Conflicts of Interest

The authors declare no conflicts of interest.

## Supporting information


**Figure S1:** Map of the study area.
**Table S1:** State‐wise summary of single‐state number of NTS outbreaks and their incidence rates (IRs) (per 1 million population) across two time periods in the United States.
**Table S2:** State‐level summary of single‐state NTS outbreak smoothed incidence rates (IRs) (per 1 million population) across two time periods in the continental contiguous United States.
**Table S3:** Incremental spatial autocorrelation analysis of NTS single‐state outbreak incidence rates in the period 2009 to 2015 across the United States.
**Table S4:** Incremental spatial autocorrelation analysis of NTS single‐state outbreak incidence rates in the period 2016 to 2022 across the United States.
**Table S5:** Summary of significant clusters of NTS single‐state outbreaks identified by various spatial analytical methods during the two study periods (2009–2015 and 2016–2022) across the continental contiguous United States, including their counts, animal categories, animal types, and exposure settings.
**Table S6:** Spatial and space–time nonsignificant clusters of animal contact‐related NTS outbreaks across.
**Table S7:** Summary of single‐state NTS outbreak clusters identified by scan statistics during the study period (2009–2022) across the continental contiguous United States, including their counts, animal categories, animal types, and exposure settings in the U.S., 2009–2022.
**Table S8:** State‐wise summary of NTS animal contact‐related single‐state outbreak counts, animal sources, and exposure settings across the US from 2009 to 2022.

## Data Availability

The data that support the findings of this study are available on request from the corresponding author. The data are not publicly available due to privacy or ethical restrictions.
