# Supplementary material for: Mapping High‐Rate Clusters of Animal Contact‐Related Human Salmonella enterica Single‐State Outbreaks in the United States, 2009–2022: A Spatial Epidemiological Approach to Inform Public Health Surveillance
Source: Zoonoses Public Health. 2026 Jun 12;73(6):502–15. doi: 10.1111/zph.70071 (PMC13535710; doi:10.1111/zph.70071)
Supplement: Supplementary file 1 [file ZPH-73-502-s001.docx]

**Supplementary Information**

**Table S1. Summary of significant clusters of NTS single-state outbreaks identified by various methods during the two study periods (2009 – 2015 and 2016 – 2022) across the continental contiguous United States, including their counts, animal categories, animal types, and exposure settings.**

| **Method** | **Cluster type** | **Period** | **State** | **Year** | **Animal type** | **Animal Category** | **Exposure Settings** | **Outbreak Count** |
| --- | --- | --- | --- | --- | --- | --- | --- | --- |
| Getis-Ord Gi* | Hotspot | 2009 – 2015 | Colorado | 2009 | Baby chick or duckling - Chick, baby chick | Birds | Other | 1 |
| Anselin local Moran’s I | High-high cluster | 2009 – 2015 |  | 2013 | Other small mammalian household pet - Mouse | Mammals | Other | 1 |
|  |  |  |  | 2014 | Other poultry - Poultry, other | Birds | Other | 2 |
|  |  |  |  | 2015 | Lizard - Bearded dragon | Reptiles | Other | 1 |
|  |  |  |  | 2015 | Turtle - Turtle | Reptiles | Other | 1 |
| Getis-Ord Gi* | Hotspot | 2016 – 2022 | Colorado | 2018 | Pig | Mammals | Other | 1 |
| Getis-Ord Gi* | Hotspot | 2016 – 2022 | Idaho | 2017 | Cattle - Cattle and Goats: Custom Slaughter workers | Mammals | Other | 1 |
| Anselin local Moran’s I | High-high cluster | 2016 – 2022 | Idaho | 2018 | Cattle - Calves | Mammals | Other | 1 |
|  |  |  | Idaho | 2018 | Other reptile - Corn snake | Reptiles | Child daycare/preschool | 1 |
|  |  |  | Idaho | 2022 | Lizard, bearded dragon | Reptiles | Residence - Single-family home | 1 |
| Getis-Ord Gi | Hotspot | 2009 - 2015 | Montana | - | - | - | - | - |
| Getis-Ord Gi | Hotspot | 2016 - 2022 | Montana | 2017 | Cattle | Mammals | Other | 1 |
| Anselin’s local Moran’s I | High-high cluster | 2016 - 2022 | Montana | 2017 | Cattle | Mammals | Other | 1 |
| Getis-Ord Gi | Hotspot | 2016 – 2022 | Nevada | - | - | - | - | - |
| Getis-Ord Gi* | Hotspot | 2016 – 2022 | South Dakota | - | - | - | - | - |
| Getis-Ord Gi* | Hotspot | 2016 – 2022 | Utah | 2016 | Other poultry - Chicken | Birds | Other | 1 |
| Anselin local Moran’s I | High-high cluster |  | Utah | 2018 | Other (specify) - Horse | Mammals | Veterinary clinic | 1 |
|  |  |  | Utah | 2019 | Cattle - baby cow | Mammals | Farm/dairy/agricultural setting | 1 |
|  |  |  | Utah | 2022 | NA | NA | Fairground | 1 |
| Getis-Ord Gi* | Hotspot | 2009 - 2015 | Wyoming | 2009 | Other small mammalian household pet - Ferret | Mammals | Other | 1 |
|  |  |  | Wyoming | 2009 | Turtle - Turtle | Reptiles | Other | 1 |
|  |  |  | Wyoming | 2012 | Pig - Pig/hog (domestic) | Mammals | Other | 1 |
|  |  |  | Wyoming | 2012 | Sheep or goats - Sheep | Mammals | Other | 1 |
| Getis-Ord Gi* | Hotspot | 2016 – 2022 | Wyoming | 2018 | Pig | Mammals | Other | 1 |
|  | High-high cluster | 2016 – 2022 | Wyoming | 2018 | Pig | Mammals | Other | 1 |
| Anselin local Moran’s I | Low-low cluster | 2009 - 2015 | Alabama | - | - | - | - | - |
| Anselin local Moran’s I | Low-low cluster | 2016 – 2022 | Alabama | 2018 | Baby chick or duckling - Buff Orpingtons | Birds | Agricultural feed store | 1 |
| Anselin local Moran’s I | Low-high outlier | 2016 – 2022 | Colorado | 2018 | Pig | Mammals | Other | 1 |
| Anselin local Moran’s I | Low-low cluster | 2009 – 2015 | District of Columbia | - | - | - | - | - |
| Anselin local Moran’s | Low-low cluster | 2009 – 2015 | Florida | - | - | - | - | - |
| Anselin local Moran’s | Low-low cluster | 2009 – 2015 | Delaware | - | - | - | - | - |
| Anselin local Moran’s I | Low-low cluster | 2009 – 2015 | Maryland | - | - | - | - | - |
| Anselin local Moran’s I | Low-high outlier | 2009 - 2015 | Montana | - | - | - | - | - |
| Anselin local Moran’s I | Low-high outlier | 2016 – 2022 | Nevada | - | - | - | - |  |
| Anselin local Moran’s I | High-low outlier | 2016 – 2022 | New Hampshire | 2017 | Turtle - pet at home | Reptiles | Other | 1 |
| Anselin local Moran’s I | Low-high outlier | 2016 – 2022 | South Dakota | - | - | - | - | - |
| Anselin local Moran’s I | Low-high outlier | 2016 - 2022 | Washington | - | - | - | - | - |
| Anselin local Moran’s I | High-low outlier | 2016 - 2022 | West Virginia | 2017 | Baby chick or duckling - Duck | Birds | Other | 1 |
|  |  |  | West Virginia | 2018 | Lizard - Gecko | Reptiles | Other | 1 |
|  |  |  | West Virginia | 2019 | NA | NA | Other | 1 |

The table includes states with their exposure sources, settings, and outbreak counts that appeared as significant hotspots, high-high clusters, high-low clusters, low-high outliers, and low-low clusters over the study period. The remaining states were insignificant.

**Table S2. Summary of single-state NTS outbreak clusters identified by scan statistics during the study period (2009 - 2022) across the continental contiguous United States, including their counts, animal categories, animal types, and exposure settings.**

| **Method** | **Cluster type** | **State** | **Outbreak count** | **Animal category** | **Animal type** | **Exposure setting** |
| --- | --- | --- | --- | --- | --- | --- |
| Purely spatial scan statistics  (2009 – 2022) | Purely spatial  (high rate)  (RR = 2.93) | Ohio | 13 | Fish, Mammals, and Reptiles | Pet fish - Fish  Dog or Puppy - Dog, puppy  Turtle - Turtle  Cattle - Cattle, calf  Other reptile - Reptile, other  Other small mammalian household pet - Guinea pig  Pig - Pig/hog (domestic)  Cattle - Cattle  Other small mammalian household pet - Hedgehog  Cattle - Calf  Cattle - Holstein and Jersey | Other  School/college/university  Farm/dairy/agricultural setting |
|  |  | West Virginia | 3 | Birds, Reptiles, and NA | Baby chick or duckling - Duck  Lizard - Gecko | Other |
|  | PS2  (low rate)  (RR = 0.10) | New York | 1 | Reptiles | Lizard - Bearded dragon | Other |
|  | PS3  (high rate)  (RR = 5.51) | Montana | 1 | Mammals | Cattle | Other |
|  |  | Idaho | 4 | Mammals and Reptiles | Lizard - Jackson's chameleon  Cattle - Cattle and Goats: Custom Slaughter workers  Cattle - Calves  Other reptile - Corn snake  Lizard, bearded dragon | Other  Child daycare/preschool  Residence - Single-family home |
|  |  | Wyoming | 5 | Mammals and Reptiles | Other small mammalian household pet - Ferret  Turtle - Turtle  Pig - Pig/hog (domestic)  Sheep or goats - Sheep  Pig | Other |
|  |  | Utah | 8 | Birds, Mammals, Reptiles, and NA | Baby chick or duckling - Chick, baby chick  Other reptile - Snake  Baby chick or duckling - Chick, baby chick  Pig - Pig/hog (domestic)  Other poultry - Chicken  Other (specify) - Horse  Cattle - baby cow | Other  Veterinary clinic  Farm/dairy/agricultural setting  Fairground |
|  |  | Colorado | 7 | Birds, Mammals, and Reptiles | Baby chick or duckling - Chick, baby chick  Other small mammalian household pet - Mouse  Other poultry - Poultry, other  Lizard - Bearded dragon  Turtle - Turtle  Pig | Other |
|  |  | South Dakota | 1 | Other poultry - Chicken | Birds | Other and school/college/university |
|  |  | Nebraska | 2 | Mammals and Birds | Hog and baby chicks | Other and child day care |
| Space-time scan statistics | ST1  2014 – 2019  (RR = 4.70)  (high rate) | Minnesota | 5 | Birds, Mammals, and Reptiles | Other poultry - Turkey  Other small mammalian household pet - Mouse  Other poultry - Turkey  Other reptile - Black roughneck monitor lizard  Other small mammalian household pet - Hedgehog  Other (specify) - Horse | Other and  Veterinary clinic |
|  |  | Wisconsin | 4 | Birds and Mammals | Baby chick or duckling - Chick, baby chick  NA  Other (specify) - cattle, sheep or goats  Other poultry - Turkey | Other and NA |
|  |  | Iowa | 3 | Birds and Mammals | Baby chick or duckling - Chick, baby chick  Other (specify) - Livestock, other  Other (specify) - Horse | Other and school/college/university |
|  |  | South Dakota | - | - | - | - |
|  |  | Nebraska | 2 | Mammals and Birds | Hog and baby chicks | Other and child day care |
|  | ST2  2013 – 2018  (RR = 5.57)  (high rate) | Ohio | 11 | Mammals and Reptiles | Dog or Puppy - Dog, puppy  Turtle - Turtle  Cattle - Cattle, calf  Other reptile - Reptile, other  Other small mammalian household pet - Guinea pig  Pig - Pig/hog (domestic)  Cattle - Cattle  Other small mammalian household pet - Hedgehog  Cattle - Calf  Cattle - Holstein and Jersey  Pig | Other, Farm/dairy/agricultural setting, and school/college/university |
|  |  | West Virginia | 3 | Birds, Reptiles, and NA | Baby chick or duckling - Duck  Lizard - Gecko | Other |
|  | ST3  2012 – 2018  (RR = 8.26)  (high rate) | Idaho | 3 | Mammals and Reptiles | Lizard - Jackson's chameleon  Cattle - Cattle and Goats: Custom Slaughter workers  Cattle - Calves  Other reptile - Corn snake | Other  Child daycare/preschool |
|  |  | Wyoming | 3 | Mammals | Pig - Pig/hog (domestic)  Sheep or goats - Sheep  Pig | Other |
|  |  | Colorado | 6 | Mammals, Birds, and Reptiles | Other small mammalian household pet - Mouse  Other poultry - Poultry, other  Lizard - Bearded dragon  Turtle - Turtle  Pig | Other |
|  |  | Utah | 5 | Birds, Mammals, and Reptiles | Other reptile - Snake  Baby chick or duckling - Chick, baby chick  Pig - Pig/hog (domestic)  Other poultry - Chicken  Other (specify) - Horse | Other  Veterinary clinic |
|  | ST4  2013 – 2017  (RR = 16.50)  (high rate) | Vermont | 2 | Birds | Baby chick or duckling - Chick, baby chick | Farm/dairy/agricultural setting  Other |
|  |  | New Hampshire | 3 | Birds, Mammals, and Reptiles | Dog or Puppy - Dog  Baby chick or duckling - Chick, baby chick  Turtle - pet at home | Other |

**Caption:** PS = Purely spatial cluster and ST = Space-time cluster

**Table S3. State-wise summary of single-state NTS outbreak numbers, and their incidence rates (IRs) (per 1 million population) across two time periods in the United States.**

| **State** | **Region** | **Outbreak numbers 2009–2015** | **Outbreak numbers 2016–2022** | **Outbreak IR 2009–2015** | **Outbreak IR 2016–2022** |
| --- | --- | --- | --- | --- | --- |
| Alabama | South | 0 | 1 | 0.00 | 0.03 |
| Alaska | West | 1 | 0 | 0.20 | 0.00 |
| Arizona | West | 1 | 1 | 0.02 | 0.02 |
| Arkansas | South | 1 | 0 | 0.05 | 0.00 |
| Colorado | West | 6 | 1 | 0.16 | 0.03 |
| Hawaii | West | 0 | 1 | 0.00 | 0.10 |
| Idaho | West | 0 | 4 | 0.00 | 0.32 |
| Illinois | Midwest | 1 | 0 | 0.01 | 0.00 |
| Indiana | Midwest | 1 | 0 | 0.02 | 0.00 |
| Iowa | Midwest | 2 | 1 | 0.09 | 0.05 |
| Kansas | Midwest | 0 | 1 | 0.00 | 0.05 |
| Kentucky | South | 2 | 0 | 0.07 | 0.00 |
| Maryland | South | 0 | 1 | 0.00 | 0.02 |
| Massachusetts | Northeast | 0 | 1 | 0.00 | 0.02 |
| Michigan | Midwest | 2 | 3 | 0.03 | 0.04 |
| Minnesota | Midwest | 2 | 4 | 0.05 | 0.10 |
| Missouri | Midwest | 2 | 0 | 0.05 | 0.00 |
| Montana | West | 0 | 1 | 0.00 | 0.14 |
| Nebraska | Midwest | 0 | 2 | 0.00 | 0.15 |
| New Hampshire | Northeast | 2 | 1 | 0.21 | 0.11 |
| New Mexico | West | 0 | 1 | 0.00 | 0.07 |
| New York | Northeast | 0 | 1 | 0.00 | 0.01 |
| North Carolina | South | 1 | 0 | 0.01 | 0.00 |
| Ohio | Midwest | 9 | 4 | 0.11 | 0.05 |
| Oregon | West | 4 | 0 | 0.15 | 0.00 |
| Pennsylvania | Northeast | 2 | 3 | 0.02 | 0.03 |
| South Carolina | South | 1 | 0 | 0.03 | 0.00 |
| South Dakota | Midwest | 1 | 0 | 0.18 | 0.00 |
| Tennessee | South | 0 | 1 | 0.00 | 0.02 |
| Texas | South | 0 | 1 | 0.00 | 0.00 |
| Utah | West | 4 | 4 | 0.20 | 0.18 |
| Vermont | Northeast | 2 | 0 | 0.46 | 0.00 |
| Virginia | South | 3 | 0 | 0.05 | 0.00 |
| Washington | West | 1 | 0 | 0.02 | 0.00 |
| West Virginia | South | 0 | 3 | 0.00 | 0.24 |
| Wisconsin | Midwest | 2 | 2 | 0.05 | 0.05 |
| Wyoming | West | 4 | 1 | 1.00 | 0.25 |

**Table S4. State-level summary of single-state NTS outbreak smoothed incidence rates (IRs) (per 1 million population) across two time periods in the continental contiguous United States.**

| State | Region | Outbreaks 2009 - 2015 | Outbreaks 2016 - 2022 | Smoothed IRs 2009 - 2015 | Smoothed IRs 2016 - 2022 |
| --- | --- | --- | --- | --- | --- |
| Alabama | South | 0 | 1 | 0.000 | 0.003 |
| Arizona | West | 1 | 1 | 0.007 | 0.004 |
| Arkansas | South | 1 | 0 | 0.003 | 0.0009 |
| Colorado | West | 6 | 1 | 0.022 | 0.006 |
| Idaho | West | 0 | 4 | 0.004 | 0.031 |
| Illinois | Midwest | 1 | 0 | 0.005 | 0.0007 |
| Indiana | Midwest | 1 | 0 | 0.005 | 0.002 |
| Iowa | Midwest | 2 | 1 | 0.005 | 0.005 |
| Kansas | Midwest | 0 | 1 | 0.006 | 0.005 |
| Kentucky | South | 2 | 0 | 0.007 | 0.0008 |
| Maryland | South | 0 | 1 | 0.003 | 0.003 |
| Massachusetts | Northeast | 0 | 1 | 0.0002 | 0.002 |
| Michigan | Midwest | 2 | 3 | 0.006 | 0.005 |
| Minnesota | Midwest | 2 | 4 | 0.009 | 0.009 |
| Missouri | Midwest | 2 | 0 | 0.004 | 0.001 |
| Montana | West | 0 | 1 | 0.005 | 0.028 |
| Nebraska | Midwest | 0 | 2 | 0.004 | 0.009 |
| New Hampshire | Northeast | 2 | 1 | 0.023 | 0.004 |
| New Mexico | West | 0 | 1 | 0.002 | 0.0065 |
| New York | Northeast | 0 | 1 | 0.0001 | 0.0025 |
| North Carolina | South | 1 | 0 | 0.003 | 0.0007 |
| Ohio | Midwest | 9 | 4 | 0.0113 | 0.006 |
| Oregon | West | 4 | 0 | 0.0138 | 0.0007 |
| Pennsylvania | Northeast | 2 | 3 | 0.0034 | 0.004 |
| South Carolina | South | 1 | 0 | 0.0028 | 0.000 |
| South Dakota | Midwest | 1 | 0 | 0.0210 | 0.0138 |
| Tennessee | South | 0 | 1 | 0.004 | 0.0009 |
| Texas | South | 0 | 1 | 0.0001 | 0.0008 |
| Utah | West | 4 | 4 | 0.026 | 0.0201 |
| Vermont | Northeast | 2 | 0 | 0.038 | 0.0021 |
| Virginia | South | 3 | 0 | 0.004 | 0.0004 |
| Washington | West | 1 | 0 | 0.0051 | 0.0004 |
| West Virginia | South | 0 | 3 | 0.006 | 0.014 |
| Wisconsin | Midwest | 2 | 2 | 0.005 | 0.005 |
| Wyoming | West | 4 | 1 | 0.070 | 0.018 |

**Table S5. Incremental spatial autocorrelation analysis of NTS single-state outbreak incidence rates in the period 2009 to 2015 across the United States**

| **Field1** | **Distance** | **Moran’s I** | **Expected I** | **Variance** | **z-score** | **p-value** |
| --- | --- | --- | --- | --- | --- | --- |
| 0 | 506559 | 0.07 | -0.021 | 0.007 | 1.112 | 0.266 |
| 0 | 588973 | 0.042 | -0.021 | 0.004 | 0.984 | 0.325 |
| 0 | 671386.9 | 0.036 | -0.021 | 0.003 | 0.988 | 0.323 |
| 0 | 753800.9 | 0.01 | -0.021 | 0.002 | 0.642 | 0.521 |
| 0 | 836214.8 | 0.012 | -0.021 | 0.002 | 0.783 | 0.434 |
| 0 | 918628.8 | -0.005 | -0.021 | 0.001 | 0.414 | 0.679 |
| 0 | 1001043 | -0.009 | -0.021 | 0.001 | 0.342 | 0.732 |
| 0 | 1083457 | -0.001 | -0.021 | 0.001 | 0.607 | 0.544 |
| 0 | 1165871 | -0.005 | -0.021 | 0.001 | 0.522 | 0.602 |
| 0 | 1248285 | -0.015 | -0.021 | 0.001 | 0.213 | 0.831 |

**Table S6. Incremental spatial autocorrelation analysis of NTS single-state outbreak incidence rates** **in the period 2016 to 2022 across the United States**

| **Field1** | **Distance** | **Moran’s I** | **Expected I** | **Variance** | **z-score** | **p-value** |
| --- | --- | --- | --- | --- | --- | --- |
| 0 | 506559 | 0.142 | -0.021 | 0.012 | 1.462 | 0.144 |
| 0 | 588973 | 0.215 | -0.021 | 0.008 | 2.686 | 0.007 |
| 0 | 671386.9 | 0.252 | -0.021 | 0.006 | 3.466 | 0.001 |
| 0 | 753800.9 | 0.168 | -0.021 | 0.004 | 2.859 | 0.004 |
| 0 | 836214.8 | 0.156 | -0.021 | 0.003 | 3.063 | 0.002 |
| 0 | 918628.8 | 0.105 | -0.021 | 0.003 | 2.39 | 0.017 |
| 0 | 1001043 | 0.117 | -0.021 | 0.002 | 2.877 | 0.004 |
| 0 | 1083457 | 0.105 | -0.021 | 0.002 | 2.844 | 0.004 |
| 0 | 1165871 | 0.057 | -0.021 | 0.002 | 1.931 | 0.053 |
| 0 | 1248285 | 0.063 | -0.021 | 0.001 | 2.282 | 0.022 |

**Table S7. State-wise summary of NTS animal contact-related single-state outbreak counts, animal sources, and exposure settings across the US from 2009 to 2022.**

| **States** | **Year** | **Animal type** | **Animal category** | **Exposure setting** | **Outbreak count** |
| --- | --- | --- | --- | --- | --- |
| Alabama | 2018 | Baby chick or duckling - Buff Orpingtons | Birds | Agricultural feed store | 1 |
| Alaska | 2011 | Turtle - Turtle | Reptiles | Other | 1 |
| Arizona | 2014 | Pig - Pigs | Mammals | Other | 1 |
| Arizona | 2016 | Other (specify) - Horse | Mammals | Hospital | 1 |
| Arkansas | 2012 | Other small mammalian household pet - Ferret | Mammals | Other | 1 |
| Colorado | 2009 | Baby chick or duckling - Chick, baby chick | Birds | Other | 1 |
| Colorado | 2013 | Other small mammalian household pet - Mouse | Mammals | Other | 1 |
| Colorado | 2014 | Other poultry - Poultry, other | Birds | Other | 2 |
| Colorado | 2015 | Lizard - Bearded dragon | Reptiles | Other | 1 |
| Colorado | 2015 | Turtle - Turtle | Reptiles | Other | 1 |
| Colorado | 2018 | Pig | Mammals | Other | 1 |
| Hawaii | 2018 | Lizard - Jackson's chameleon | Reptiles | Other | 1 |
| Idaho | 2017 | Cattle - Cattle and Goats: Custom Slaughter workers | Mammals | Other | 1 |
| Idaho | 2018 | Cattle - Calves | Mammals | Other | 1 |
| Idaho | 2018 | Other reptile - Corn snake | Reptiles | Child daycare/preschool | 1 |
| Idaho | 2022 | Lizard, bearded dragon | Reptiles | Residence - Single-family home | 1 |
| Illinois | 2012 | Turtle - Turtle | Reptiles | Other | 1 |
| Indiana | 2009 | NA | NA | NA | 1 |
| Iowa | 2013 | Baby chick or duckling - Chick, baby chick | Birds | Other | 1 |
| Iowa | 2014 | Other (specify) - Livestock, other | Mammals | Other | 1 |
| Iowa | 2016 | Other (specify) - Horse | Mammals | School/college/university | 1 |
| Kansas | 2018 | Lizard - Bearded dragon | Reptiles | Other | 1 |
| Kentucky | 2012 | Turtle - Turtle | Reptiles | Other | 1 |
| Kentucky | 2013 | Lizard - Bearded dragon | Reptiles | Other | 1 |
| Maryland | 2016 | NA | NA | Other | 1 |
| Massachusetts | 2017 | Other poultry - Chicken | Birds | Other | 1 |
| Michigan | 2013 | Other (specify) - Horse | Mammals | Other | 1 |
| Michigan | 2014 | Turtle - Turtle | Reptiles | Other | 1 |
| Michigan | 2016 | Other poultry - Chicken | Birds | Other | 1 |
| Michigan | 2016 | Other small mammalian household pet - Hedgehog | Mammals | Other | 1 |
| Michigan | 2019 | Cattle | Mammals | Other | 1 |
| Minnesota | 2014 | Other poultry - Turkey | Birds | Other | 1 |
| Minnesota | 2014 | Other small mammalian household pet - Mouse | Mammals | Other | 1 |
| Minnesota | 2016 | Other poultry - Turkey | Birds | Other | 1 |
| Minnesota | 2016 | Other reptile - Black roughneck monitor lizard | Reptiles | Other | 1 |
| Minnesota | 2016 | Other small mammalian household pet - Hedgehog | Mammals | Other | 1 |
| Minnesota | 2019 | Other (specify) - Horse | Mammals | Veterinary clinic | 1 |
| Missouri | 2010 | Pet fish - Fish | Fish | Other | 1 |
| Missouri | 2015 | NA | NA | Other | 1 |
| Montana | 2017 | Cattle | Mammals | Other | 1 |
| Nebraska | 2017 | Pig - Hog | Mammals | Other | 1 |
| Nebraska | 2019 | Baby chick or duckling - Chicks | Birds | Child daycare/preschool | 1 |
| New Hampshire | 2013 | Dog or Puppy - Dog | Mammals | Other | 1 |
| New Hampshire | 2014 | Baby chick or duckling - Chick, baby chick | Birds | Other | 1 |
| New Hampshire | 2017 | Turtle - pet at home | Reptiles | Other | 1 |
| New Mexico | 2018 | Other poultry - duckling | Birds | Other | 1 |
| New York | 2016 | Lizard - Bearded dragon | Reptiles | Other | 1 |
| North Carolina | 2009 | Other small mammalian household pet - Guinea pig | Mammals | Other | 1 |
| Ohio | 2009 | Pet fish - Fish | Fish | Other | 1 |
| Ohio | 2011 | Pet fish - Fish | Fish | Other | 1 |
| Ohio | 2013 | Dog or Puppy - Dog, puppy | Mammals | Other | 1 |
| Ohio | 2013 | Turtle - Turtle | Reptiles | Other | 1 |
| Ohio | 2014 | Cattle - Cattle, calf | Mammals | Farm/dairy/agricultural setting | 1 |
| Ohio | 2014 | Other reptile - Reptile, other | Reptiles | Other | 1 |
| Ohio | 2014 | Other small mammalian household pet - Guinea pig | Mammals | Other | 1 |
| Ohio | 2014 | Pig - Pig/hog (domestic) | Mammals | Other | 1 |
| Ohio | 2015 | Cattle - Cattle | Mammals | Farm/dairy/agricultural setting | 1 |
| Ohio | 2016 | Other small mammalian household pet - Hedgehog | Mammals | Farm/dairy/agricultural setting | 1 |
| Ohio | 2018 | Cattle - Calf | Mammals | School/college/university | 1 |
| Ohio | 2018 | Cattle - Holstein and Jersey | Mammals | Farm/dairy/agricultural setting | 1 |
| Ohio | 2018 | Pig | Mammals | Farm/dairy/agricultural setting | 1 |
| Oregon | 2009 | Baby chick or duckling - Chick, baby chick | Birds | Farm/dairy/agricultural setting | 1 |
| Oregon | 2009 | Other reptile - Snake | Reptiles | Other | 1 |
| Oregon | 2014 | NA | NA | NA | 2 |
| Pennsylvania | 2011 | Turtle - Turtle | Reptiles | Other | 1 |
| Pennsylvania | 2012 | Turtle - Red-eared slider turtle | Reptiles | Other | 1 |
| Pennsylvania | 2018 | Turtle | Reptiles | Other | 1 |
| Pennsylvania | 2019 | Baby chick or duckling - ducklings | Birds | Other | 1 |
| Pennsylvania | 2020 | Baby chick or duckling | Birds | Agricultural feed store | 1 |
| South Carolina | 2015 | Baby chick or duckling - Chick, baby chick | Birds | Other | 1 |
| South Dakota | 2010 | Other poultry - Chicken | Birds | School/college/university | 1 |
| Tennessee | 2017 | Baby chick or duckling - Baby chicks | Birds | School/college/university | 1 |
| Texas | 2018 | Baby chick or duckling - duckling | Birds | Other | 1 |
| Utah | 2010 | Baby chick or duckling - Chick, baby chick | Birds | Other | 1 |
| Utah | 2012 | Other reptile - Snake | Reptiles | Other | 1 |
| Utah | 2014 | Baby chick or duckling - Chick, baby chick | Mammals | Other | 1 |
| Utah | 2014 | Pig - Pig/hog (domestic) | Mammals | Other | 1 |
| Utah | 2016 | Other poultry - Chicken | Birds | Other | 1 |
| Utah | 2018 | Other (specify) - Horse | Mammals | Veterinary clinic | 1 |
| Utah | 2019 | Cattle - baby cow | Mammals | Farm/dairy/agricultural setting | 1 |
| Utah | 2022 | NA | NA | Fairground | 1 |
| Vermont | 2015 | Baby chick or duckling - Chick, baby chick | Birds | Farm/dairy/agricultural setting | 1 |
| Vermont | 2015 | Baby chick or duckling - Chick, baby chick | Birds | Other | 1 |
| Virginia | 2009 | Turtle - Turtle | Reptiles | Child daycare/preschool | 1 |
| Virginia | 2011 | Amphibian - Frogs | Amphibians | Child daycare/preschool | 1 |
| Virginia | 2012 | Bird, not including poultry - Vulture | Birds | Other | 1 |
| Washington | 2010 | Baby chick or duckling - Chick, baby chick | Birds | Other | 1 |
| West Virginia | 2017 | Baby chick or duckling - Duck | Birds | Other | 1 |
| West Virginia | 2018 | Lizard - Gecko | Reptiles | Other | 1 |
| West Virginia | 2019 | NA | NA | Other | 1 |
| Wisconsin | 2014 | Baby chick or duckling - Chick, baby chick | Birds | Other | 1 |
| Wisconsin | 2015 | NA | NA | NA | 1 |
| Wisconsin | 2017 | Other (specify) - cattle, sheep or goats | Mammals | Other | 1 |
| Wisconsin | 2019 | Other poultry - Turkey | Birds | Other | 1 |
| Wyoming | 2009 | Other small mammalian household pet - Ferret | Mammals | Other | 1 |
| Wyoming | 2009 | Turtle - Turtle | Reptiles | Other | 1 |
| Wyoming | 2012 | Pig - Pig/hog (domestic) | Mammals | Other | 1 |
| Wyoming | 2012 | Sheep or goats - Sheep | Mammals | Other | 1 |
| Wyoming | 2018 | Pig | Mammals | Other | 1 |
| NA | 2009 | Baby chick or duckling - Chick, baby chick | Birds | Child daycare/preschool | 1 |
| NA | 2009 | Baby chick or duckling - Chick, baby chick | Birds | Other | 2 |

**Table S8. Spatial and space-time nonsignificant clusters of animal contact-related NTS outbreaks across**

**the U.S., 2009 – 2022.**

| **Cluster** | **Model type** | **Number of States** | **Time frame** | **Population** | **N/C** | **E/C** | **O/E** | **LR** | **RR** | **p-value** |
| --- | --- | --- | --- | --- | --- | --- | --- | --- | --- | --- |
| 1. | Purely spatial | 2 | _ | 1975331 | 5 | 0.90 | 5.58 | 4.57 | 5.82 | 0.176 |
| 2. | Purely spatial | 2 | _ | 19433159 | 2 | 8.82 | 0.23 | 4.10 | 0.21 | 0.266 |
| 3. | Purely spatial | 2 | _ | 15020643 | 5 | 6.82 | 0.29 | 2.48 | 0.28 | 0.830 |
| 4. | Purely spatial | 4 | _ | 20597223 | 5 | 9.35 | 0.53 | 1.32 | 0.51 | 0.997 |
| 1. | Space-time | 2 | 2016 – 2022 | 19451717 | 0 | 4.54 | 0 | 4.65 | 0 | 0.77 |
| 2. | Space-time | 1 | 2009 – 2015 | 19616550 | 0 | 4.43 | 0 | 4.53 | 0 | 0.82 |
| 3. | Space-time | 2 | 2015 – 2021 | 19433159 | 0 | 4.42 | 0 | 4.51 | 0 | 0.83 |
| 4. | Space-time | 3 | 2009 – 2014 | 16459085 | 0 | 3.10 | 0 | 3.15 | 0 | 0.999 |

^1^N/C – Number of cases, E/C – Expected cases, O/E – Observed/Expected, LR – Likelihood ratio, RR – Relative Risk.

**Figure S1. Map of the study area.**
